# Moose–tree interactions: rebrowsing is common across tree species

**DOI:** 10.1186/s12898-017-0122-3

**Published:** 2017-04-04

**Authors:** Karen Marie Mathisen, Jos M. Milner, Christina Skarpe

**Affiliations:** 1 Department of Forestry and Wildlife Management, Faculty of Applied Ecology and Agricultural Sciences, Inland Norway University of Applied Sciences, Pb 400, 2418 Elverum, Norway; 2 School of Biological Sciences, University of Aberdeen, Tillydrone Avenue, Aberdeen, AB24 2TZ UK

**Keywords:** Tolerance, Compensatory growth, Palatability, Regrowth, Brooming, Accumulated browsing, Height, Alces alces

## Abstract

**Background:**

Plant strategies to resist herbivory include tolerance and avoidance. Tolerance strategies, such as rapid regrowth which increases the palatability of new shoots, can lead to positive feedback loops between plants and herbivores. An example of such a positive feedback occurs when moose (*Alces alces*) browse trees in boreal forests. We described the degree of change in tree morphology that accumulated over time in response to repeated browsing by moose, using an index of accumulated browsing. We evaluated whether accumulated browsing could predict the probability and extent of current browsing across woody species in a Norwegian boreal forest, and how our accumulated browsing index related to changes in tree height, shoot availability and shoot size.

**Results:**

The probability and extent of current browsing increased with the degree of accumulated browsing in all tree species. Plants highly modified by previous browsing were the most attractive, with no indication of decreased preference with repeated browsing over time. The preference for previously browsed trees is most likely driven by increased relative availability of shoots within browsing height and maybe increased palatability. This response to previous browsing was general for both preferred and avoided forage species, in both conifers and deciduous trees.

**Conclusions:**

Our results suggest that the adaptation for rapid regrowth after browsing does not reduce herbivory on trees. Rather, our results indicate that plant responses to browsing increase the probability of future herbivory. This response could potentially lead to higher plant mortality where cervid populations are maintained at stable high densities and has implications for plant population dynamics and forestry practices.

**Electronic supplementary material:**

The online version of this article (doi:10.1186/s12898-017-0122-3) contains supplementary material, which is available to authorized users.

## Background

Plant–herbivore interactions are important drivers of population and ecosystem dynamics, and affect ecosystem processes such as nutrient cycling and succession [1]. Furthermore, an understanding of small scale plant–herbivore interactions is important for understanding larger scale dynamics [2]. Herbivore selection within and between individual plants can affect large scale processes by, for example, changing the rate or direction of succession depending on the successional stage of selected species [3].

Plants have evolved a diverse set of strategies to avoid or tolerate predation from herbivores [4]. Plant responses to herbivory are context dependent, varying with plant species [5], competition [6], season [7–9], time since previous browsing [10] and frequency and intensity of browsing [5, 11], as well as the plant part browsed [8] and habitat productivity [5, 10]. Avoidance strategies include having thorns and small leaves, and responding to herbivory by increasing these traits to reduce intake rate and bite size, thus deterring herbivory on the same plant [12]. Similarly, constitutive or induced chemical defenses deter herbivory by affecting taste, reducing digestibility or by being toxic to the herbivore [13]. These chemical or structural defense responses reduce the probability of herbivory, creating a negative feedback loop between the plant and the herbivore. By contrast, plant tolerance strategies involve responses to herbivory such as increased growth rate, increased shoot size and increased resource allocation from root to shoot, allowing plants to compensate for herbivory without deterring herbivores [14, 15]. Tolerance responses may increase the risk of future herbivory if plants produce larger or more vigorous shoots that have a higher nutrient concentration or lower concentration of defense compounds [16]. As many herbivores feed preferentially on such plants or plant modules [17], this can create a positive feedback loop between plants and herbivores [16].

Our study focuses on plant tolerance responses and the positive feedback driven by browsing and re-browsing. How plants respond to previous browsing may in turn affect future browse selection and biomass removal, with implications for plant species composition and dynamics. An example of a tolerance response that increases the probability of future browsing occurs when browsing on leading shoots reduces the apical dominance of leading meristems, an adaptation to plant competition [18, 19]. Removal of dominant meristems reduces nutrient competition with apical shoots, and in turn benefits the browser by increasing shoot production at lower, readily available, heights [8, 20]. In addition, rapid regrowth reduces the synthesis of secondary metabolites leading to more palatable shoots for browsers [16].

A positive feedback between plant and herbivore has been observed in several studies of re-browsing by moose (*Alces alces*) in the boreal forest ecosystem [21–24]. Both the probability of a tree being browsed and browsing pressure may increase with previous browsing [21, 22, 25, 26]. In addition, bite size may increase as a response to increased shoot size and palatability [9, 26]. The pattern of moose responses to previous browsing may also differ between trees with different growth patterns. Biomass production of browsed birch (*Betula pubescence* and *B. pendula*) may increase with moderate moose browsing, whilst biomass production of Scots pine (*Pinus sylvestris*) decreases [5]. Differences in responses to browsing may be linked to determinate versus indeterminate growth patterns, and to different sites of nutrient storage in deciduous and evergreen trees [27–30].

Repeated browsing generally reduces tree height growth in both coniferous and deciduous species [22]. The number of shoots available per tree has been observed to decrease with browsing in birch and pine [31]. However, browsing often increases the production of branched shoots in birch [32], as well as the number of shoots available to moose in rowan [33], hence the overall availability of shoots in deciduous trees may either increase or decrease with previous browsing. Shoot morphology and chemistry may also change in response to browsing. Annual shoot size has been observed to either increase [6, 20, 34] or decrease [6, 33] in response to moose browsing depending on plant species and time scale, which may affect the size of available bites on previously browsed trees [9, 26]. Browsing may affect the concentration of nutrients and secondary compounds in shoots. Increases in structural carbohydrates may be required to support the growth of large compensatory shoots but reduces their digestibility [7]. In contrast, regrowth from browsed shoots in willow (*Salix phylicifolia*) was less toxic and more digestible than growth from unbrowsed shoots [34].

As current browsing is related to previous browsing through positive feedback loops between plants and herbivores, an index of previous browsing is expected to be a strong predictor of current browsing [35, 36]. In this study, we have used an index of accumulated browsing [37] which describes the degree of change in tree structure that accumulates over time in response to repeated browsing by moose. We quantified the degree of accumulated browsing occurring across tree species in young boreal forest managed for timber production in south-eastern Norway and investigated three specific questions: (i) whether accumulated browsing could predict the probability and extent of current browsing; (ii) how current browsing differed in response to accumulated browsing between trees with different growth pattern and (iii) how the accumulated browsing index reflected changes in shoot availability, tree height and bite sizes.

Within species, we predicted that moose would respond to accumulated browsing by increasing their selection of trees with higher levels of previous browsing (i.1). Consequently, we expected an increase in the number of recently browsed shoots (i.2) and bite diameter (i.3) as the level of previous browsing increased. Given the higher capability of deciduous trees for compensatory growth and the production of more palatable biomass after browsing [5], we expected moose preference for birch (indeterminate growth) over pine (determinate growth) to increase as accumulated browsing increased (ii). Hence, we expected that browse selection (ii.1) and intensity (ii.2 and ii.3) would be higher for birch than pine at high levels of accumulated browsing. Based on previous work [31, 32], we predicted that the number of shoots available per tree would increase with accumulated browsing for birch, but decrease for pine (iii.4). We also predicted that tree height would decrease (iii.5), and shoot size would increase (iii.6: diameter; iii.7: length) with increasing accumulated browsing.

## Methods

The aim for this study was to investigate how accumulated browsing in the past can affect current moose browsing on young trees. We quantified the degree of accumulated browsing occurring across tree species in young boreal forest managed for timber production in south-eastern Norway.

### Study areas

**Fig. 1:**
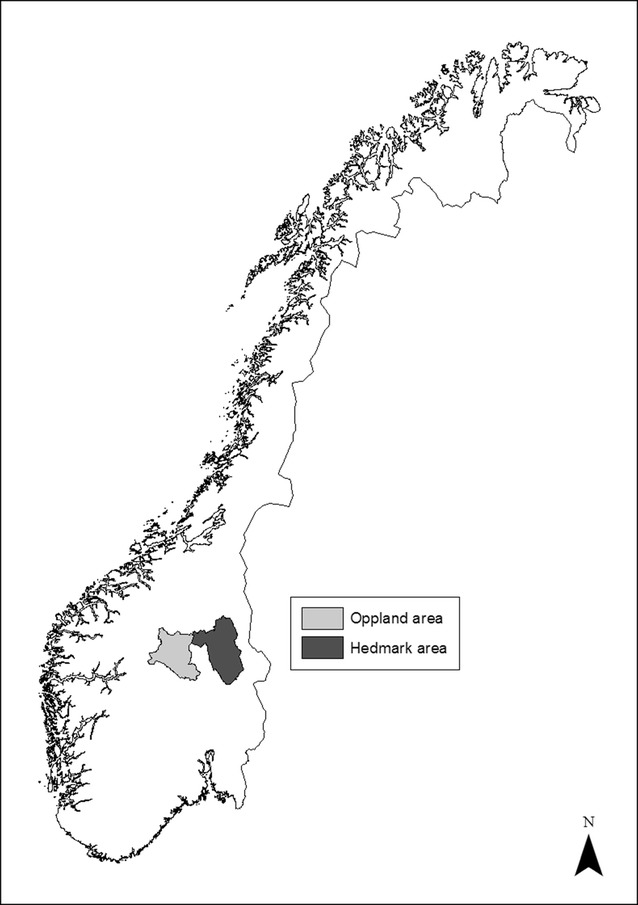
Map of Norway with study area indicated. Young Scots pine (*Pinus sylvestris*) stands were surveyed for moose browsing in the indicated areas in Hedmark (2010) and Oppland (2011)

This study was carried out in the counties of Oppland and Hedmark in south-eastern Norway (~61°N, 11°E, Fig. 1). Within these study areas, forest stands were located in Stor-Elvdal, Åmot and Rendalen municipalities in Hedmark, and Gausdal, Sør-Fron, Nord-Fron, Sel and Vågå municipalities in Oppland. The vegetation was primarily boreal forest [38] below the commercial timberline, managed for Scots pine and Norway spruce (*Picea abies*) timber and pulp production. Pine stands regenerate naturally, so the young pine stands in this study contained commercial and non-commercial tree species, both of which provide forage for moose. The site productivity index for pine in both areas was low to medium [39]. Stands were dominated by Scots pine, Norway spruce, and downy birch interspersed with silver birch, grey alder (*Alnus incana*), rowan (*Sorbus aucuparia*), aspen (*Populus tremula*), willows (*Salix* spp.) and juniper (*Juniperus communis*). The field layer vegetation was dominated by dwarf shrubs such as *Vaccinium* spp. The Hedmark study area was situated between 250 and 1100 m above sea level with 30-year mean summer (May–September) and winter (October–April) temperatures of 10.6 °C and −5.8 °C, respectively, in the valley bottom. The 30-year mean annual precipitation was 628 mm and the mean snow depth (October–April) was 39 cm [40]. The Oppland study area had a slightly higher elevation (515–920 m a s l), with a mean annual precipitation of 564 mm, winter temperature of −5.0 °C, summer temperature of 7.0 °C (30-year mean) and snow depth of 67 cm (average for the last 10 years). The study area was characterized by valleys and mountains and in both cases; moose tend to migrate down to the less snowy valley bottoms during winter. In the Hedmark area, winter density was approximately 1.3 moose per km^2^ [41], for Oppland there were no records on moose density.

### Field survey

We selected young forest stands based on age and tree species composition. In Hedmark, young forest stands of pure and mixed Scots pine were identified from satellite maps of forest stands from the Norwegian Forest and Landscape Institute [42]. Spruce dominated stands were excluded, as spruce is rarely eaten by moose [25, 43]. As supplementary feeding of moose is common in this area, only stands >1 km from supplementary feeding stations were included, to avoid confounding effects on browsing. Previous studies have shown that supplementary feeding sites affect moose browsing intensity at a local scale (<1 km from feeding sites) but not at the landscape scale [44, 45]. In Oppland, young stands were identified from forestry maps from Statskog (Norwegian state-owned forest company), the main landowner in the study area. All stands were visited to confirm that they were dominated by Scots pine and had trees of the desired height (0.5–5 m), with live branches within moose browsing height ≤3 m [26]. The resulting sample consisted of 69 stands in Hedmark and 42 stands in Oppland.

Forest stands were surveyed in June–July 2010 in Hedmark and May–June 2011 in Oppland using 50 m^2^ circular plots. In Hedmark, four plots were surveyed within each stand, distributed 20 m from the centre point in each cardinal direction (N, S, E, and W). In Oppland, six plots were surveyed within each stand, laid out systematically in a grid using ArcGIS software. Each plot was at least 20 m from the edge, and at least 20 m from each other with the distance between plots increasing with the size of the stand.

Within all plots, moose pellet groups from the previous winter were counted to provide an index of moose density [46]. We classified plot vegetation type according to Moen et al. [38], based on the dominant field-layer vegetation species, and used this as an index of forest productivity (Additional file 1) ranging from low to medium to high [38, 39, 47].

Within each plot, we counted all trees taller than 0.5 m and assessed them for moose browsing (in total 12,565 trees were measured, see Table 1 for sample sizes per species). Trees below 0.5 m height were assumed to be covered by snow during winter, when most browsing occurs. For each measured tree, we recorded the total number of shoots (defined as twigs >1 cm long) from the last growing season, within moose browsing height (0.5–3 m). We classified shoots as either moose browsed or unbrowsed. Tree height was measured to the nearest 10 cm for trees ≤5 m. On each tree, we measured the diameters of 3–5 browsed shoots (if present) at the point of browsing (hereafter called bite diameter). We assigned each tree a qualitative accumulated browsing index (hereafter abbreviated to ABI, [37, 44], to describe the cumulative effect of previous browsing on tree structure (i.e. excluding browsing during the most recent winter). The scores were as follows: ABI 0 = no previous browsing, ABI 1 = previous browsing visible but the tree structure was mainly unchanged, ABI 2 = previous browsing had visibly modified the structure of the tree (such as crooked stem, increased branching), ABI 3 = previous browsing had strongly modified the structure of the tree (i.e. multiple leader stems, hedged state, brooming). Trees that had modified structure due to other causes and showed no old bite marks, were classified as ABI 0. Old bite marks were usually visible on leading shoots (ABI 2 and 3), broom-shaped shoots (ABI 3), or on side shoots (ABI 1). The ABI incorporated a time effect as trees in class 3 showed signs of repeated browsing over multiple years, whilst trees in class 1–2 may have only been browsed in 1 year.

**Table 1:** Number of measured trees in the accumulated browsing index (ABI) categories for all tree species

Species	ABI 0	ABI 1	ABI 2	ABI 3	Total sum
Scots pine *Pinus sylvestris*	1797	1195	1623	708	5323
Downy birch *Betula pubescens*	1104	877	939	304	3224
Norway spruce *Picea abies*	1444	60	39	*4*	1547
Silver birch *Betula pendula*	284	201	368	43	896
Willows *Salix* sp.	118	41	255	100	514
Juniper *Juniperus communis*	330	45	75	14	464
Rowan *Sorbus aucuparia*	15	33	143	220	411
Aspen *Populus tremula*	*9*	*1*	42	70	122
Grey alder *Alnus incana*	24	29	11	*0*	64
Total sum	5125	2482	3495	1463	12,565

*ABI 0 * no previous browsing by moose.* ABI 1*  previously browsed, but structure of the tree has not changed,* ABI 2*  previous browsing has caused a change in tree structure,* ABI 3*  strongly modified structure due to previous browsing. Combinations with low sample size (<10 trees) are indicated by italic

To evaluate the relationship between ABI and shoot diameter and length (predictions iii.6–7), we sampled shoots of all tree species, except spruce and alder (the least browsed species), in Hedmark. We sampled 1087 shoots from 554 randomly selected trees, by stopping every 500 m along a forest road, and walking 50 m into the forest stand alternating between left and right side of the road, and selecting the closest 3 trees of each species found. We measured diameter and length on 3 randomly selected shoots per tree, by choosing the closest shoot in each height class above ground, if available (0.5–1.0 m, 1.1–1.5 m, 1.6–2.0 m). The diameter was measured at the base of the shoot to the nearest 0.1 mm and the length was measured from the base of the shoot to the base of the terminal bud to the nearest 0.1 cm. Only 12 of the recorded shoots were branched so these were subsequently excluded from analyses.

### Statistical analysis

The effects of ABI on current moose browsing and tree morphology were analyzed in R 3.1.0 [48], using mixed models within the nlme [49] and lme4 [50] packages. The models and explanatory variables required to test each prediction are shown in Table 2. To analyze whether browse selection of individual trees increased with ABI (prediction i.1 and ii.1), we used a generalized linear mixed model, with the occurrence of browsing fitted as a binomial response variable (0/1) and a logit-link function. Predictions i.2–iii.7 were analyzed using linear mixed models, verifying assumptions of normality with residual plots. Numbers of shoots browsed (prediction i.2) and shoots available (prediction iii.4) were log_n_-transformed, other response variables were normally distributed. ABI and site productivity indices were fitted as categorical variables. We also controlled for variation in the variables pellet group density and forest productivity, by fitting them as additional fixed effects. We used plot identity within stand identity and study area (Oppland/Hedmark) as nested random intercept terms to account for unbalanced sample sizes between different plots and stands, and to control for non-independence within plots and stands.

**Table 2:** Overview of variables included in linear and generalised linear mixed models to test each prediction

Prediction	Response variable	Predictor variables	Random intercept
i.1	Probability of browsing (0/1)	ABI, moose, prod	Area/stand/plot
i.2	Ln(browsed.shoots)	ABI, moose, prod, ln(av.shoots + 1)	Area/stand/plot
i.3	Bite diameter (mm)	ABI, moose, prod	Area/stand/plot
ii.1	Probability of browsing (0/1)	ABI*sp, moose, prod	Area/stand/plot
ii.2	Ln(browsed.shoots)	ABI*sp, moose, prod, ln(av.shoots + 1)	Area/stand/plot
ii.3	Bite diameter (mm)	ABI*sp, moose, prod	Area/stand/plot
iii.4	Ln(av.shoots +1)	ABI*ln(tree height), prod	Area/stand/plot
iii.5	Tree height (standardized)	ABI*stand height, prod	Area/stand/plot
iii.6	Shoot diameter	ABI*sp, height above ground	Plot/tree ID
iii.7	Shoot length	ABI*sp, height above ground	Plot/tree ID

Predictions i.1–ii.3 investigate the moose response (current browsing) to accumulated browsing (ABI), while predictions iii.4–7 investigate the tree’s morphological response to previous browsing. Prediction i.1–3 were analysed separately for each individual tree species. Prediction ii.1–3 and iii.4–5 were analysed for birch and pine only, because they provided sufficient data. For prediction iii.6–7 all tree species were grouped together, excluding spruce and alder due to insufficient data

*Sp*  species,* moose*  moose pellet groups,* prod*  productivity index from vegetation type,* av. shoots*  available shoots in browsing height (0.5–3 m)

Sample sizes among tree species and ABI categories varied greatly (0-1960) and were unbalanced (Table 1). Consequently models that included the interaction species*ABI would not converge. Therefore, predictions i.1–3 were analyzed individually for all tree species. Then we investigated the interaction between ABI and species separately for Scots pine and downy birch for which we had sufficient data across ABI categories. We used Scots pine and downy birch as examples of different growth forms (evergreen with determinate growth versus deciduous with indeterminate growth) to test prediction ii, the effect of interaction between ABI and growth form on current moose browsing (predictions ii.1–3). We evaluated the effect of fixed effects on response variables using a comparison of likelihoods between nested models in a backward step selection procedure [51]. We only present the effects of accumulated browsing on current browsing and morphology as this was the focus of our study.

A small number of trees above 5 m in height were present in the stands (e.g. seed trees, trees left after logging), but these were excluded from all analysis as we wanted to focus on trees with live branches within browsing height for moose (0.5–3 m). For analyses of the occurrence of browsing (i.1 and ii.1), shoots available (iii.4) and tree height (iii.5), all trees ≤5 m were included. For the analyses of shoots browsed (i.2 and ii.2) and bite diameter (i.3 and ii.3), only trees browsed by moose the current winter were included. The relationship between ABI and tree morphology [number of shoots available (iii.4), tree height (iii.5)] was investigated further for Scots pine and downy birch. For the analysis of effects of ABI on tree height, the height of each tree was subtracted from the average stand height to account for differences among stands in height development, and the analysis was performed on standardized tree height. The effect of the interaction between ABI and stand height on relative tree height was included to see how ABI was related to height development among trees. Number of shoots browsed was positively related to number of shoots available within browsing height, so the interaction between ABI and shoots available was included in the analysis of shoots browsed (i.2 and ii.2), to investigate if the slope between shoots available and browsed changed with ABI. Tree height was also strongly positively correlated with number of shoots available at browsing height, so the interaction between tree height and ABI was included in this analysis (iii.5), to investigate if the slope between tree height and shoot production changed with ABI.

## Results

**Fig. 2:**
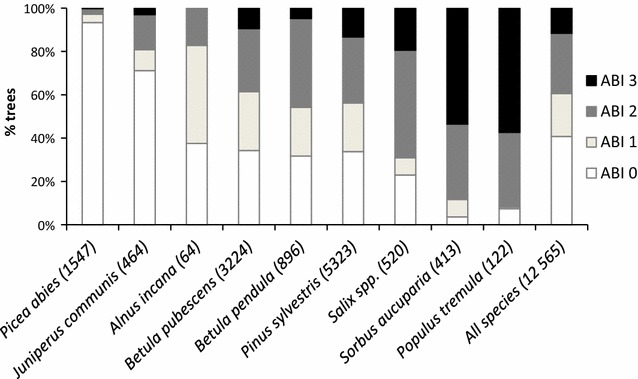
Percent (%) of trees per species in each accumulated browsing index (ABI) category, sorted from low to high values. ABI 0 = no previous browsing by moose. ABI 1 = previously browsed, but structure of the tree has not changed, ABI 2 = previous browsing has caused a change in tree structure, ABI 3 = strongly modified structure due to previous browsing. Sample size per species added in brackets. See Table 1 for common names

The degree of accumulated browsing differed markedly between tree species (Fig. 2). In highly preferred tree species such as rowan and aspen, 80–90% of the trees were categorized as structurally modified or heavily modified by previous browsing (ABI 2 and 3, Fig. 2). In contrast, <5% of spruce trees had previously been browsed. The two birch species and pine were intermediate, with around 40–45% of trees in classes ABI 2 and 3. Across all species, 40% of trees were previously unbrowsed by moose.

### Effects of accumulated browsing on current browsing of all tree species

**Fig. 3:**
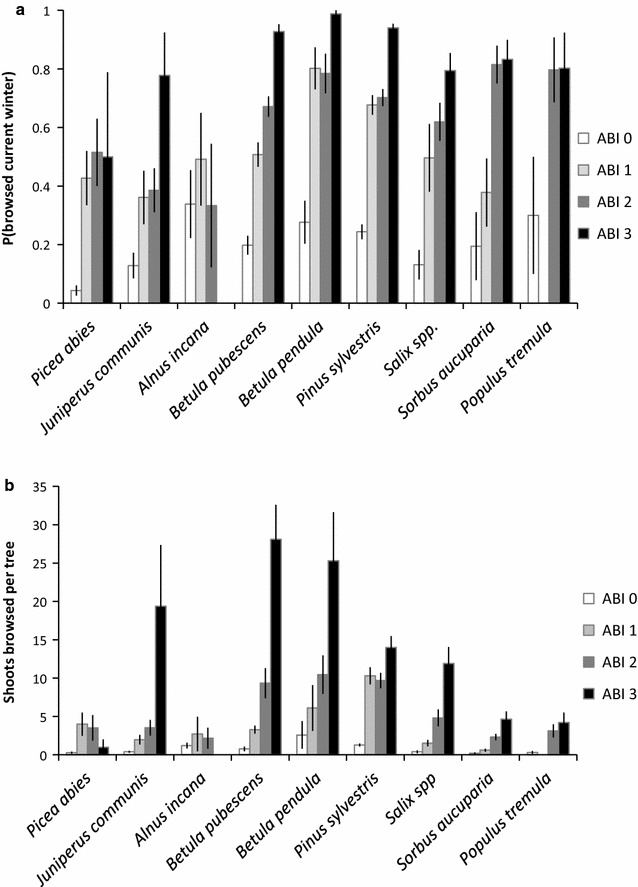
Moose browsing in the current winter in relation to accumulated browsing (see Fig. 2 for definition) in the past for woody plant species in young boreal forest. **a** Proportion of trees browsed by moose in the current winter (mean ± SE). **b** Number of browsed shoots per tree (mean ± SE) on browsed trees. See Table 1 for sample size and common names

As predicted (i.1 and i.2), ABI was a positive predictor of current moose browsing. Both the probability of current browsing (i.1, Fig. 3a) and the number of recently browsed shoots per tree (i. 2, Fig. 3b) increased significantly with increasing ABI across all species (Table 3). However, the moose response to the degree of accumulated browsing differed between tree species. For juniper, birches, pine and willow, trees strongly modified (ABI 3) by previous browsing had the highest probability of being re-browsed, whilst for rowan and aspen, modified (ABI 2) and strongly modified (ABI 3) trees had an equal probability of rebrowsing (Fig. 3a). For spruce, all previously browsed trees had an equal probability of being re-browsed, but only 4 trees were classified as ABI 3 (Fig. 3a). For alder there were no trees in ABI 3, and in general there was little data to evaluate this species. The number of browsed shoots per tree showed a strong increase in highly modified trees (ABI 3) relative to other classes in juniper, birches and willow (Fig. 3b). In general, and contrary to prediction i.3, bite diameter showed no relationship with ABI (model 3, Table 3). Rowan and aspen were exceptions but small samples sizes within the unbrowsed classes meant these results should be interpreted with caution (see Tables 1, 3).

**Table 3:** Results from linear and generalized mixed models (Table 2) analyzing the effects of the fixed effects; accumulated browsing index (ABI), moose density (pellet groups) and site productivity (Additional file 1) on moose browsing in the current winter for all tree species in young forest stands ≤5 m high

Species	Response variable	Acc. browsing Ind. ABI (dF = 3)	Moose density (dF = 1)	Productivity (dF = 2)
Pine	Probability of browsing (0/1)	*χ* ^*2*^ = *936.01, p* < *0.001*	*χ* ^*2*^ = *5.18, p* = *0.023*	*χ* ^2^ = 0.99, p = 0.609
	Ln(browsed.shoots)	*L* = *190.46, p* < *0.001*	*L* = *17.85, p* < *0.001*	L = 1.62, p = 0.445
	Bite diameter (mm)	L = 6.29, p = 0.098	L = 0.61, p = 0.435	L = 4.49, p = 0.106
Downy birch	Probability of browsing (0/1)	*χ* ^*2*^ = *431.44, p* < *0.001*	*χ* ^*2*^ = *3.99, p* = *0.046*	*χ* ^2^ = 0.28, p = 0.868
	Ln(browsed.shoots)	*L* = *283.45, p* < *0.001*	*L* = *12.59, p* < *0.001*	L = 5.679, p = 0.058
	Bite diameter (mm)	L = 8.12, p = 0.506	L = 1.63, p = 0.202	*L* = *8.17, p* = *0.017*
Silver birch	Probability of browsing (0/1)	*χ* ^*2*^ = *130.58, p* < *0.001*	*χ* ^2^ = 0.74, p = 0.391	*χ* ^2^ = 0.35, p = 0.552
	Ln(browsed.shoots)	*L* = *83.97, p* < *0.001*	*L* = *15.48, p* < *0.001*	L = 1.63, p = 0.201
	Bite diameter (mm)	L = 1.60, p = 0.660	L = 1.08, p = 0.299	L = 0.98, p = 0.321
Rowan	Probability of browsing (0/1)	*χ* ^*2*^ = *46.00, p* < *0.001*	*χ* ^2^ = 1.39, p = 0.239	*χ* ^*2*^ = *5.54, p* = *0.019*
	Ln(browsed.shoots)	*L* = *22.93, p* < *0.001*	L = 0.79, p = 0.375	L = 0.18, p = 0.675
	Bite diameter (mm)	*L* = *11.35, p* = *0.010*	L = 2.01, p = 0.156	L = 0.22, p = 0.634
Willows	Probability of browsing (0/1)	*χ* ^*2*^ = *97.22, p* < *0.001*	*χ* ^2^ = 3.69, p = 0.055	*χ* ^2^ = 0.03, p = 0.859
	Ln(browsed.shoots)	*L* = *26.47, p* < *0.001*	*L* = *7.20, p* = *0.007*	L = 0.01, p = 0.942
	Bite diameter (mm)	L = 4.61, p = 0.203	L = 0.47, p = 0.492	L = 0.73, p = 0.392
Aspen	Probability of browsing (0/1)	*χ* ^*2*^ = *14.48, p* < *0.001*	*χ* ^2=^2.34, p = 0.126	*χ* ^2^ = 1.44, p = 0.486
	Ln(browsed.shoots)	*L* = *6.82, p* = *0.033*	L = 0.54, p = 0.461	L = 3.93, p = 0.140
	Bite diameter (mm)	*L* = *15.67, p* < *0.001*	*L* = *4.54, p* = *0.033*	L = 1.04, p = 0.560
Juniper	Probability of browsing (0/1)	*χ* ^*2*^ = *46.36, p* < *0.001*	*χ* ^2^ = 0.45, p = 0.504	*χ* ^2^ = 0.77, p = 0.379
	Ln(browsed.shoots)	*L* = *20.98, p* < *0.001*	L = 3.65, p = 0.056	L = 0.03, p = 0.866
	Bite diameter (mm)	L = 4.69, p = 0.196	L = 2.11, p = 0.147	L = 2.96, p = 0.086
Spruce	Probability of browsing (0/1)	*χ* ^*2*^ = *92.74, p* < *0.001*	*χ* ^2^ = 1.24, p = 0.266	*χ* ^*2*^ = *9.74, p* = *0.008*
	Ln(browsed.shoots)	L = 6.51, p = 0.089	L = 0.21, p = 0.645	L = 0.00, p = 0.953
	Bite diameter (mm)	L = 1.93, p = 0.587	L = 1.61, p = 0.205	L = 2.13, p = 0.145
Grey alder^a^	Probability of browsing (0/1)	*χ* ^2^ = 4.83, p = 0.089	*χ* ^2^ = 0.46, p = 0.497	*χ* ^*2*^ = *4.50, p* = *0.034*

For each fixed effect, nested models including/excluding the variable were compared in a likelihood ratio test, and the Likelihood ratio (L), dF and p value (<0,05 in italic) is presented for linear models, and a similar Chi square (χ^2^) test for the binomial model for browsing probability. For sample sizes and scientific names, see Table 1. ABI 0 = no previous browsing by moose. ABI 1 = previously browsed, but structure of the tree has not changed, ABI 2 = previous browsing has caused a change in tree structure, ABI 3 = strongly modified structure due to previous browsing

^a^For grey alder, the data on browsed trees were to scarce to analyze shoots browsed and bite diameter

### Differences in current browsing responses between Scots pine and downy birch

**Fig. 4:**
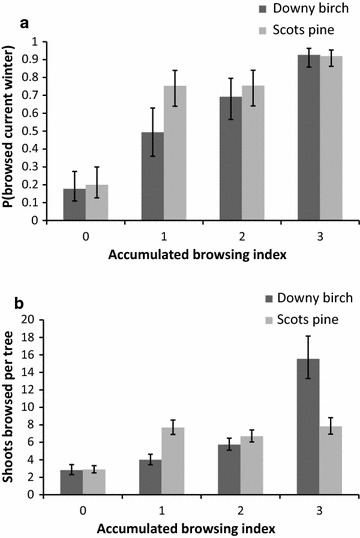
Moose browsing in the current winter in relation to accumulated browsing (see Fig. 2 for definition) in the past for downy birch (*Betula pubescens*) and Scots pine (*Pinus sylvestris*). **a** Browsing probability (browsed vs unbrowsed trees). **b** Number of browsed shoots per tree on browsed trees

The relationship between the probability of moose browsing in the current winter and ABI differed between Scots pine and downy birch trees (interaction—species*ABI: χ^2^ = 43.86, df = 3, p < 0.001). Although the probability of current browsing increased with the degree of ABI for both species, the observed pattern was not as predicted in ii.1. Current browsing of the lightly browsed class was much higher for pine than birch, while at high levels of accumulated browsing there was little difference in current browsing probability between species (Fig. 4a).

**Fig. 5:**
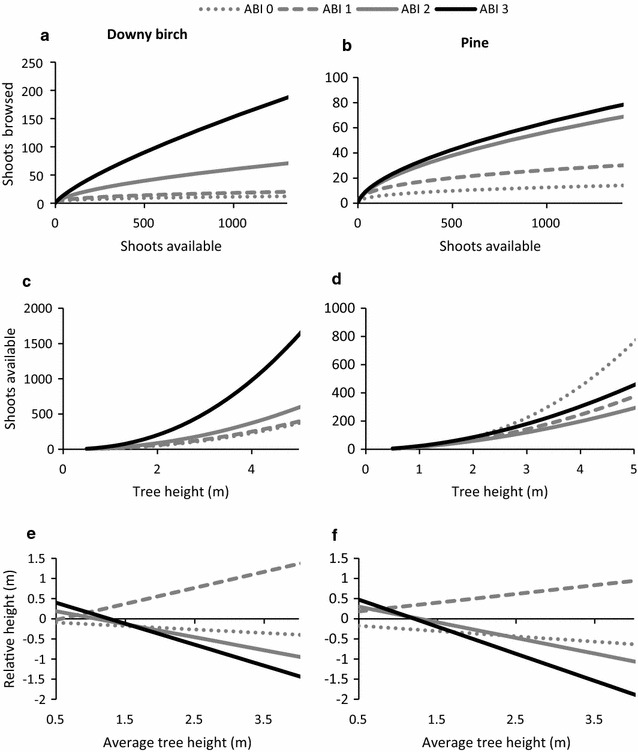
Responses to varying degrees of accumulated browsing (ABI—definition in Fig. 2) in downy birch (*Betula pubsecencs*, *left*) and pine (*Pinus sylvestris*, *right*). **a**, **b** The relationship between number of shoots browsed in the current winter by moose per tree and the number of shoots available within browsing height. **c**, **d** The relationship between tree height and shoots available in browsing height (0.5–3.0 m) for trees of height 0.5–5 m with different degree of ABI in the past. **e**, **f** Effect of the interaction between average tree height in the stand and ABI on the relative tree height (height-average tree height)

The number of shoots browsed per tree also differed between birch and pine in relation to ABI (interaction—species*ABI: χ^2^ = 150.18, df = 3, p < 0.001). As predicted (ii.2), in highly modified trees (ABI 3) the number of recently browsed shoots was higher for birch than pine, but, contrary to expectation, the opposite was true of lightly modified trees (ABI 1, Fig. 4b). Hence, for pine, the main effect was a difference in the number of browsed shoots between previously browsed and unbrowsed trees, but for birch the number of shoots browsed increased gradually with increasing ABI. As the number of recently browsed shoots was positively related to the number of shoots available, we investigated the interaction between ABI and available shoots on the number of moose browsed shoots for pine and downy birch separately. The interaction was significant for both species (pine: L = 77.20, df = 3, p < 0.001, birch: L = 119.57, df = 3, p < 0.001) such that the number of browsed shoots increased more steeply in relation to available shoots for higher levels of ABI (Fig. 5a, b). However, the relationship increased gradually between ABI classes in pine whereas in birch it was steeper for strongly modified trees (ABI 3) than other classes (Fig. 5a, b).

Contrary to prediction ii.3, there was no effect of the interaction between ABI and species on bite diameter (model 3 interaction—tree type*ABI: χ^2^ = 1.07, df = 3, p = 0.785). However, bite diameter was larger in pine (3.98 ± 0.15 mm) than birch (2.16 ± 0.15 mm; χ^2^ = 1346.28, df = 3, p < 0.001).

### Morphological plant responses to previous browsing

The number of shoots available within browsing height increased with tree height for both pine and downy birch. In pine, there was also a significant interaction between ABI and tree height affecting the number of shoots available (L = 245.08, df = 3, p < 0.001). Previously unbrowsed trees had more shoots available per height increment than previously browsed trees (Fig. 5d), indicating that accumulated browsing led to reduced shoot production in pine. For downy birch, the effect of the interaction between tree height and ABI on number of shoots available within browsing height was also significant but less strong and in the opposite direction (L = 7.96, df = 3, p = 0.047). Therefore, as predicted (iii.4), the number of shoots per height increment increased with ABI in birch but decreased for pine (Fig. 5c, d).

There was also an effect of the interaction between average tree height per stand and ABI on relative tree height for both pine (L = 286.94, df = 3, p < 0.001) and downy birch (L = 155.59, df = 3, p < 0.001; Fig. 5e, f). The direction of the effect was similar in both species. Trees that had not been previously browsed by moose tended to be shorter than the average tree in the stand, while trees that had been lightly browsed but showed no change in structure (ABI 1) tended to be taller than the average tree. Trees where browsing had modified (ABI 2), or strongly modified (ABI 3) tree structure, tended to be shorter than the average tree in agreement with prediction iii.5 (Fig. 5e, f). The difference in height among ABI classes became apparent above 1 m height, and increased with increasing stand height.

The diameter of annual shoots was not affected by the interaction between species and ABI (L = 25.18, df = 18, p = 0.120) or by ABI class alone (L = 2.34, df = 3, p = 0.504). Similarly shoot length did not vary significantly with the interaction between species and ABI (L = 20.78, df = 18, p = 0.290) or with ABI alone (L = 6.04, df = 3, p = 0.110). Predictions that shoot diameter (iii.6) and shoot length (iii.7) would increase with ABI were therefore not supported.

## Discussion

### Moose and tree responses to accumulated browsing

Moose browsing, in terms of browsing probability and number of shoots browsed, increased with the accumulation of past browsing in all tree species studied in our boreal forest system. Our study is the first to show that this relationship occurred in both highly preferred (rowan, aspen, willow) and less preferred (spruce and alder) browse species [25, 52], and in both deciduous and coniferous trees, suggesting it may be a general pattern. Highly modified trees (ABI 3) were the most preferred, with no indication of decreased preference with repeated browsing over time. However, we found no support for our prediction that bite size would increase with accumulated browsing. Previous studies have shown a tendency for rebrowsing on the same tree [21–26, 34, 35, 53], which could have been caused by selection for larger or more palatable shoots, or higher browse availability at foraging height [20, 54]. Our lack of a bite size effect indicates that height, availability of shoots and maybe palatability may be more important than shoot size.

### Moose reinforce height variation among trees

Both birch and pine trees with a high level of accumulated browsing (2 and 3) were of below average height and relatively shorter than trees with previous light browsing. In addition, moose preferred highly modified trees to unbrowsed trees of a similar height, indicating that height was not the only factor behind greater moose preference for modified trees. Apical shoots were frequently browsed, with top shoot browsing reported on 59% of pines and 66% of birches in Hedmark [44]. This breaking of apical dominance would reduce vertical growth. Apical dominance is an adaption to ensure rapid growth in height when competing for light in a dense forest [18]. Removal of the apical meristem, and release of lateral meristems from hormonal control, may lead to compensatory growth responses that make browsed trees more palatable [8, 18, 20, 54, 55]. For example, mobilization of stored carbon for regrowth means less carbon is available for production of secondary defense compounds [16]. In addition, rapid regrowth is considered to be a response mechanism enabling plants to grow out of browsing height [14, 28, 56]. However, if regrowth is insufficient to escape browsing height, it makes the plant more attractive to herbivores the following year by creating a high availability of forage within browsing height [8, 29].

Hence adaptations to plant competition may in turn reduce plant competitiveness under certain conditions. Other studies have also found that repeated browsing reduces height growth in boreal tree species [22]. Energy constraints may limit regrowth over multiple years and repeated pruning of a woody plant may reduce its carbon reserves, preventing both compensatory growth and production of carbon–based defense compounds [28]. This is particularly likely in boreal forest with medium–low productivity [5, 57], as was typical of our study area.

By contrast, lightly browsed trees were taller than average, whilst unbrowsed trees were shorter. There are several possible explanations for this. Unbrowsed trees may have become established in the stand later and had their subsequent growth suppressed by competition from taller trees. According to the plant vigor hypothesis [17], moose prefer fast-growing trees with larger and more nutritious shoots [58] so suppressed trees may be less attractive. Lightly browsed trees may have become established in the stand early on and developed rapidly in height due to low competition, or having been browsed once, responded with sufficient compensatory growth to escape above browsing height if browsing pressure was low [6, 9]. As differences in height between trees with different degrees of accumulated browsing increased with the average stand height, lightly browsed trees were likely to form the future stand canopy.

Our results show that accumulated browsing by moose leads to or reinforces height variation within young stands [22], and could reduce plant competition for lightly browsed trees. As these grow above browsing height, browsing patches of trees with a high degree of accumulated browsing will eventually be overtopped and outcompeted for light by other trees, leaving patches of strongly modified dead trees under the canopy. By creating habitat for insects and fungi in managed forests that otherwise lack this type of habitat, herbivore selection for previously browsed trees may increase habitat heterogeneity [2], with positive effects for conservation of biodiversity.

#### Shoot availability increased in birch, but decreased in pine

We predicted that deciduous trees would have a higher capability for compensatory regrowth than conifers [27]. This was supported by an increase in shoot availability with increasing accumulated browsing in birch, but a reduction in pine. In birch, the increase in shoot availability may be due to increased shoot sprouting at lower meristems on previously browsed trees [32, 58] and/or increased branching. Production of branched shoots could also lead to a larger number of bites being available for moose within browsing height. The higher availability of birch shoots likely explained the greater number of shoots browsed by moose on highly modified birch than pine trees (ABI 3). The reduction in shoot availability with increased accumulated browsing in pine has been shown previously [59] and can be explained by the deterministic growth pattern of pine. Each year a whorl of shoots is added to the main axis and all branch axes, and new shoots form by elongation of terminal buds, formed in the previous season [8]. Therefore in pine, the ability to produce new shoots after browsing is restricted to a few meristems. In birch the growth form is more flexible, allowing dormant and short shoots along the stem and branches to convert into long-shoots and new shoots to form after browsing [58]. In addition, as nutrients are stored in the needles in pine during winter but in the roots and stems in birch [27], pine suffers proportionally higher losses due to winter browsing, and has fewer resources available for compensatory growth.

#### Shoot and bite size did not increase with accumulated browsing

We found no evidence of larger shoots on previously browsed trees. Although some other studies have shown a decrease in shoots size in response to winter browsing [7, 33], most previous studies have shown increases in shoot size [9, 10, 23, 26, 32, 34], or needle size in pine [24], which we did not measure. The discrepancy may have arisen because we studied multiple species in natural forest stands with low productivity and recurring browsing, whilst most other studies focused on only one species in one season, and, in some cases, were simulated browsing experiments. Plants that have been repeatedly browsed over several years may have depleted resources, and be less able to compensate for browsing by producing large shoots, particularly in low productivity sites. Edenius et al. [10] found an initial increase in pine shoot size in the first year of simulated moose browsing, but a decrease in shoot size in the second and third years. Low nutrient availability in our study area may have limited compensatory growth of large shoots [5]. Furthermore, small differences in which plant part is removed can create different responses [8], showing that response patterns are complex.

Moose have previously been reported to browse on larger shoots of moderately than lightly browsed trees [26]. The greater preference for previously browsed trees was therefore partly explained by the selection of large shoots [60, 61] in order to maximize net energy gain [62]. We predicted that moose bite diameters would increase with accumulated browsing, but did not find this relationship in any tree species. In our study, this was most likely explained by the fact that we found no increase in shoot diameter with accumulated browsing. Therefore, our results do not support the idea that moose selected previously browsed trees in order to gain larger bites.

#### Plant chemical responses

We have not investigated plant chemical composition in this study. However, the observed preference for trees with high accumulated browsing could be caused by increased nutrient concentration or reduced concentration of plant secondary compounds. Previous studies have found that the carbon demands of regrowth reduces production of secondary defense compounds in previously browsed trees [7, 16, 28, 34], while increased nutrient concentration can occur because of the reduced number of meristems [18, 19]. Moose selection of winter browse is known to be negatively related to the concentration of specific phenolics in *Salix phylicifolia*, and concentrations were lower in previously browsed shoots [63]. Nitrogen concentration in needles has also been found to be slightly higher in browsed than unbrowsed pine trees [24], but most studies of effects on birch show a neutral or negative impact of winter browsing on nutrient concentration of shoots [9]. Plant chemical responses to rebrowsing require further research across a range of species and environmental conditions.

### Implications of rebrowsing

Both preferred and avoided tree species, and tree species with different growth form responded to rebrowsing in similar ways, supporting the hypothesis that some tolerance traits are a general adaptation against disturbance (drought, fire, herbivory), and not specifically an adaptation to resist herbivory [64]. In the past, evolutionary pressure from competition has likely been stronger than evolutionary pressure from herbivory. However, over recent decades densities of large herbivores have increased dramatically [65, 66] with the consequence that increased attractiveness to herbivores due to compensatory growth may become maladaptive. Indeed, most studies conclude that rebrowsing reduces flowering, seed production and long-term plant survival, although in some cases it may increase biomass at smaller spatial or temporal scales [26, 67, 68]. In other cases, producing attractive shoots for herbivores may be adaptive at the individual plant level. For modular organisms, it has been suggested that producing shoots of differing palatability to herbivores can be a two-level strategy. By offering some attractive shoots to herbivores, reproduction and growth are concentrated on other highly defended shoots, which then escape herbivory [34]. Further monitoring of the long-term survival and fitness of plants with different degrees of rebrowsing, is needed to answer these questions.

Rebrowsing may be beneficial for moose, as they can return to the same place every year, and browse on previously browsed trees that now have an increased availability of palatable shoots within browsing height. It has even been suggested that rebrowsing by moose may be an example of resource regulation, with the food quality being improved for the accompanying next generation [64]. Rebrowsing by one herbivore may also facilitate other herbivores in the community, as browsing at the foraging height of a tall herbivore may increase shoot production lower in the canopy [36]. As birch shows better regrowth than pine, and birch leaves are important summer forage for moose, rebrowsing may increase the relative availability of summer forage for moose in more heavily browsed stands. However, although patch quality and relative forage availability may improve, total forage biomass generally decreases with browsing over the longer term [5, 33], potentially increasing searching time. In addition, rebrowsing might increase the speed of vegetation succession [69], leading to shading of forage patches and reduced forage availability.

#### Management implications

At a stand level, rebrowsing leads to the non-random distribution of browsing and increases the variation in height growth among trees [22]. This may have both positive and negative impacts on timber production, as moose carry out the thinning operations for the forester but create an uneven distribution of trees and may not achieve the desired stand density. We recommend forest managers keep preferred rebrowsed trees within the stand, rather than removing them in pre-commercial thinning, in order to reduce browsing on unbrowsed timber crop trees.

In natural systems, large unmanaged herbivore populations tend to naturally fluctuate between periods of high and low density [70], allowing trees to regenerate in pulses [35]. However, often game management aims to maintain large stable populations which might keep preferred browse species in a hedged state, with knock-on effects for biodiversity. Furthermore, the positive feedback loop between plants and herbivores may lead to a more rapid depletion of forage resources than expected, as patches of heavily browsed trees become overtopped by less browsed trees. In order to co-manage forage resources and cervid populations sustainably, it would be beneficial to include the effect of rebrowsing in models of forest development to predict forage availability for cervids.

## Conclusions

We found that preferences for all tree species increased with previous browsing. Similar feedback loops between woody plants and browsers have been observed not only in low productive boreal forest, but also in temperate forest and semi-arid savanna [11, 37, 71]. Herbivore selection among woody plants seems to be strongly related to their response to previous browsing, and may be a general feature of tree-browser interactions in forest communities. In our study, all tree species showed a similar direction of compensatory response to browsing, leading to increased herbivore use with increasing accumulated browsing. This indicates the absence of induced qualitative defenses in response to browsing, and occurs regardless of preference among tree species. Our results therefore support the view that plant responses to browsing attract rather than deter future browsing. This type of tolerance response may potentially be maladaptive for the plant, at least in areas with low productivity and high herbivore browsing pressure. If cervid populations are managed at stable high densities, consequences for plant population dynamics should be expected, together with possible evolutionary effects on plant defenses.

## Additional file

**Additional file 1.** Table of definitions of productivity classes from vegetation types.

## Abbreviations

ABI: accumulated browsing index
ABI 0: no previous browsing
ABI 1: previous browsing visible but the tree structure was mainly unchanged
ABI 2: previous browsing had visibly modified the structure of the tree (such as crooked stem, increased branching)
ABI 3: previous browsing had strongly modified the structure of the tree (i.e. multiple leader stems, hedged state, brooming)
