# Supplementary material for: Moose–tree interactions: rebrowsing is common across tree species
Source: BMC Ecol. 2017 Apr 4;17:12. doi: 10.1186/s12898-017-0122-3 (PMC5381076; doi:10.1186/s12898-017-0122-3)
Supplement: Supplementary file 1 — Additional file 1. Table of definitions of productivity classes from vegetation types. [file 12898_2017_122_MOESM1_ESM.pdf]

Supplementary material for the manuscript “Moose – tree interactions: rebrowsing is common across tree species”. Description of vegetation types in relation to forest productivity. Vegetation types were used as an indication of forest productivity. Productivity class (low/medium/high) were uses in the analysis.

**Vegetation type**	**Forest productivity description (from (Fremstad 1997))**	**Productivity class used in analysis**	**Number of plots**
Lichen	Very low	Low	179
Heather	Low to medium	Medium	62
Berries	Medium to low	Medium	99
Bilberry	Low to medium	Medium	30
Small fern	Medium to high	High	1
Low herbs	High	High	150
Tall herbs	High	High	10
